# Applications of Hydroxyapatite-Based Polymeric Scaffolds in Bone Tissue Engineering: An Update

**DOI:** 10.34172/apb.43818

**Published:** 2024-10-16

**Authors:** Nazanin Amiryaghoubi, Rana Jahanban Esfahlan

**Affiliations:** ^1^Kidney Research Center, Tabriz University of Medical Sciences, Tabriz, Iran.; ^2^Department of Medical Biotechnology, Faculty of Advanced Medical Sciences, Tabriz University of Medical Sciences, Tabriz, Iran.

**Keywords:** Hydroxyapatite, Scaffold, Bone tissue engineering

## Abstract

Bone organ is comprised of an organic and inorganic environment, in which the collagen element and the mineral part are structured into spongy constructions. Hydroxyapatite (HAp) is the chief inorganic constituent of human bone. HAp is extensively utilized in bone tissue regeneration for its biocompatibility and a rising number of investigators are discovering ways to recover the physical belongings and biological roles of HAp. However, this biomimetic material indicates poor mechanical strength, for example, low tensile and compressive strength, which offer it inappropriate for bone tissue engineering. For this point, HAp is frequently utilized in a mixture with diverse polymers to increase their mechanical strengths and the general function of the implantable biomaterials advanced for orthopedic usage. In this review, we attempt to contribute a brief and inclusive outline of HAp-based natural and synthetic polymer materials to strengthen structures and their applications in bone tissue regeneration.

## Introduction

**Scheme 1:**
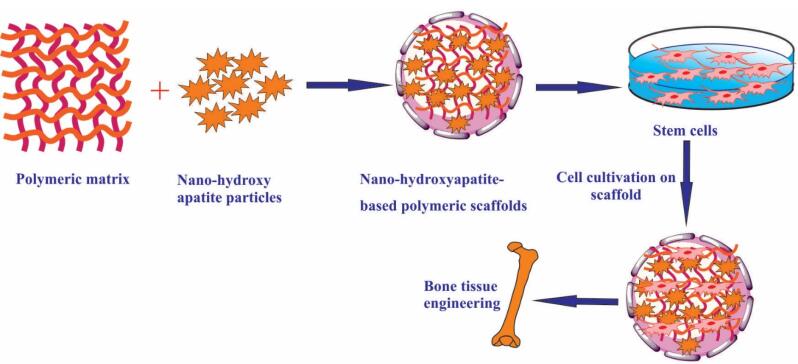
Schematic representation of n-HAp-based polymeric scaffolds for bone tissue regeneration

 Hydroxyapatite [Ca_10_(PO_4_)_6_(OH)_2_; (HAp)] is the evolving greatest bioceramic and most usual calcium phosphate ceramic, which is commonly utilized in numerous biomedical usages, mostly in orthopedics and dentistry because of its similar resemblances with a mineral component of bone and teeth.^1-4^ It has been recognized that HAp nanoparticles (NPs) can meaningfully enhance the biocompatibility and osteogenesis of fabricated biomaterials.^5^ HAp is a biomaterial that can be taken out of ordinary wastes.^6^ HAp has been extensively utilized in the restoration of hard organs, and general usages contain bone renovation, and bone intensification, besides covering implants or performing as fillers in bone and teeth. It has been advocated that nano-HAp (nHAp) could be a perfect selection owing to its worthy biocompatibility and bone assimilation capability.^7^ Bone tissue regeneration is a substitute method to produce bone utilizing biomaterials and cells. HAp indicated worthy biocompatibility and osteoconductivity.^8-13^ Aside from its benefits, HAp indicates a poor mechanical property which restricts its wide usage, particularly as an implant material in bone tissue regeneration. So, it is vital to incorporate the other polymeric or reinforced nanomaterials in scaffolds-based HAp to recover mechanical properties (Scheme 1).^14^ Also, optimization of different parameters involving HAp concentration or composite biomaterials for ideal scaffold formation can be attempted *in silico* by Molecular Dynamic Stimulation.^15^ Recent reviews explored the role of bovine HAp^16^ and Hap in three-dimensional printing.^17^ In this review, we highlight some usages of HAp-based polymeric scaffolds employed to produce a perfect platform for bone regeneration.

## Scaffolds-based natural polymer-HAp in bone tissue engineering

###  Scaffold-based chitosan- HAp in bone tissue engineering

**Figure 1:**
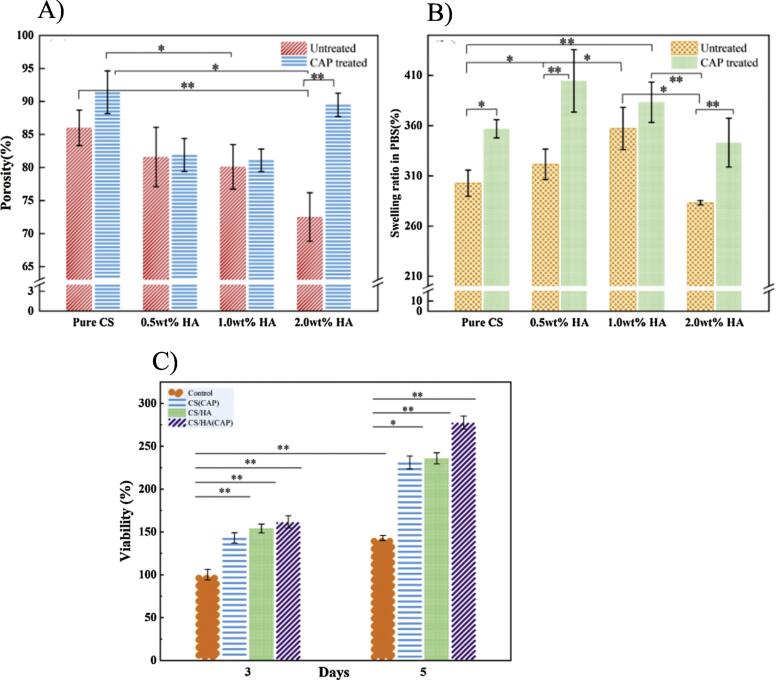
A) The porosity ratio of freeze-dried composites with various HAp amounts before and later CAP handling, B) the swelling percentage of the platform, and C) the viability of MC3T3-E1 cells on composites after 3 and 5 days, assessed by MTT test (*P < 0.05, **P < 0.01). Reproduced with permission from Li et al.^33^ Copyright Elsevier (2021)

 Chitosan (CS) is a bioactive polymer with a varied diversity of requests because of its practical possessions including antibacterial behavior, biocompatibility, easiness of alteration, and biodegradability.^18-21^ CS an arising polymer, is attractive for tissue-regeneration usages because of its great nontoxicity and hydrophilicity.^22^ CS scaffolds are osteoconductive and capable of increasing bone development in both in vitro and in vivo environment.^23-31^ CS has long been reflected as unique of the greatest striking ordinary biopolymers for bone tissue regeneration due to its organizational resemblance to the glycosaminoglycan originating in bone and outstanding mechanical strengths.^32^ For example, in a study, CS united with HAp was inserted into a composite braid, and a spongy construction platform was attained by freeze drying and cold atmospheric plasma (CAP) method. Consequences revealed that HAp incorporation able the central of the platform with macroscopic hole dimensions of 80–180 μm, and CAP usage able the shell of the platform with microscopic pore dimensions ≤ 10 μm. All platforms displayed extraordinary sponginess and swelling percentages of ≥ 80 % and ≥ 300 %, respectively (Figure 1A and 1B). The composite with a hierarchical pore construction indicated worthy mechanical strengths and two times the degradation degree. In the synergistic result of HAp and CAP conduct, composites accomplished 277.6 % cell growth relative to the CS platform, alone (Figure 1C). Generally, this technique was possible for arranging bone platforms with hierarchical spongy construction for probable bone tissue regeneration.^33^ Ali et al fabricated the CS, hydroxypropyl methyl cellulose (HPMC), HAp, and Lemon grass oil (LGO) founded platforms by freeze gelation technique for bone regeneration. An escalation in LGO content contributed to enhanced porosity along with feasibly reduced mechanical properties and diminished density. Based on the cytotoxicity of CS-HPMC-HAp-LGO results, no toxic sign of MC3T3-E1 cells cultured on the CS-HPMC-HAp-LGO scaffold was observed. The research outcomes demonstrated that all composite of CS-HPMC-HAp-LGO with a spongy system indicated meaningful antibacterial properties.^34^ In a study, high-compactness polyethylene (HDPE)–CS–HAp mixture sequences with changing content of HAp were fabricated. Positron annihilation lifetime spectroscopy assay exhibited that the free pores were made in the array of ∼115.8 Å^3^. MC3T3 E1 cell lines indicated suitable cell growth on the improved organizations. The existence of micropores along with CS and HAp enhanced cell proliferation in the fabricated scaffolds. The HDPE–CS scaffold with 5 wt% palm oil (HP5) was adjusted because of its worthy miscibility of the constituents in the organization and heightened thermal firmness. The structural test revealed no phase departure among the fillers and matrix. Consequently, the prepared hybrid biocomposite indicated possible bone biomedical implantations.^35^

 The nHAp-impregnated CS/κ-carrageenan (nHAp/CS/κ-CGN) nanocomposite was fabricated over the co-precipitation method and was compared with nHAp/CS nanocomposite for bone restoration ability. Based on the in vitro biomineralization assay, enhanced formation of apatite coating on nHAp/CS/κ-CGN nanocomposite was observed as relative to nHAp/CS scaffold. The MG-63 cell culture assay exhibited superior cell growth when cultivated in the existence of nHAp/CS/κ-CGN nanocomposite relative to nHAp/CS. Moreover, suitable swelling rate, enriched protein adsorption, and promising degradation speed were also perceived for the nHAp/CS/κ-CGN platform. So, nHAp/CS/κ-CGN nanocomposite could be a superior selection for bone tissue regeneration.^36^ Tarmidzi et al studied the influence of CS in bone implantation composite selection from a mixture of HAp and magnesium (Mg) on its properties including compressive strength and crystallinity. HAp/Mg powder was produced through the sol-gel technique by reaction of Ca (NO_3_)_2_.4H_2_O, MgCl_2_.6H_2_O, and (NH_4_)_2_HPO_4_ in a basic situation. HAp/Mg was sintered at 600 °C for 2 hours. The incorporation of CS included decreasing composite crystallite size and compression strength. Extreme compression strength was attained at 11%w/v CS amount with 14.45 MPa with crystallite dimension 23.14 nm. This consequence was equivalent to the maximum strength informed of trabecular bone.^37^ Chen et al fabricated nacre-copied HAp/CS/gelatin (HAp/CS/Gel) coated platforms and combined substance P (SP) peptides for bone restoration. The HAp/CS/Gel covered platforms indicated prodigious flexure strength of 11.42 MPa owing to a brick-and-mortar construction. The biocompatible constituents and SP peptides in the HAp/CS/Gel healing platforms enhanced the distribution and growth of mesenchymal stem cells (MSCs). Particularly, the integration of SP peptides prompted MSC osteogenesis and extracellular matrix (ECM) calcium deposition. Rabbit knee subchondral flaw models additionally confirmed that the HAp/CS/Gel-SP coated platforms endorsed in vivo subchondral bone restoration. Therefore, the mixture of nacre-imitative bone implantations and healing drugs might develop a striking approach for bone restoration.^38^

###  Scaffold-based gelatin- HAp in bone tissue engineering

 Gel is utilized in tissue engineering works due to its biocompatibility and biodegradability possessions.^39-41^ The Gel is reflected in an appropriate selection to mimic the ECM owing to its functional groups and the probability of producing 3D platforms with spongy construction.^42-44^ In a study, the polycaprolactone (PCL)/Gel/HAp NPs (PGH) platform was effectively produced over the electrospinning method. The morphological observation showed comparatively bead-identical fibers and the fiber diameter, pore dimension, and sponginess stayed at 616 nm, 9 μm, and 86 %, respectively. Based on the results, the PGH platform was biocompatible and it enriched the stem cells’ attachment, growth, and differentiation meaningfully. Based on the in vivo results, the stem cell-cultured platforms could meaningfully hasten the healing of the cranial bone flaw.^45^ In a study, porous Gel/HAp (G/H) as an environment was prepared to offer exceptional mechanical strengths with restorable properties, and human placental extracts (hPE) inserted in the platform have been utilized as bioactive constituents. The in vitro consequences showed that the growth of G/H and G/H/hPE was 351.1 ± 13.3% and 430.9 ± 8.7% after 14 days. The expression of osteogenic genes (alkaline phosphatase (ALP), bone morphogenetic proteins (BMP2), osteopontin (OPN), and osteocalcin (OCN)) of human adipose stem cells (hASCs) cultivated in the G/H/hPE platform was 3.4-fold, 3.9-fold, 1.7-fold, 2.4-fold, and: 4.8-fold after 21 days.^46^ The hollow channel platforms were comprised of Gel and Gel-altered nHAp produced by extrusion molding for bone tissue regeneration. Based on the findings, the fabricated Gel-modified nHAp were nanorod-similar fragments with a length of 30∼50 nm and a width of 5∼15 nm. The NPs contained crystalline HAp particles with a dimension of less than 10 nm and shapeless phases. The synthesized hollow channel platforms indicated considerably better mechanical strengths relative to the porous platforms. The axial compressive strength of the hollow channel platform with sponginess of 51.3 ± 5.2% could reach 25 ± 1.4 MPa, which could encounter the mechanical property necessity as a graft. Furthermore, the hollow channel platform showed appropriate in vitro weight loss and bioactivity. These consequences confirmed the possibility of utilizing Gel/Gel-altered nHAp composite platforms with hollow networks for bone regeneration.^47^ In a study, a bulk agarose–Gel hydrogel was mineralized with electrophoresis, and the mineralized hydrogel was laden with minocycline to attain the agarose–Gel–HAp–minocycline nanocomposite. The prepared scaffold indicated a huge Brunauer–Emmett–Teller exterior area of 44.4518 m^2^ g^−1^ and an extraordinary sponginess of 76.9%. The assimilation of minocycline contributed to a constant antibiotic release, impeding the development of Staphylococcus aureus ended 2 weeks in vitro. Open to rabbit bone marrow mesenchymal stem cells (BMSCs), the scaffold indicated worthy biocompatibility. Additionally, the biomaterial could efficiently increase bone restoration in a critical-dimension rabbit cranial flaw model in vivo. Based on the results, the prepared scaffold demonstrated suitable biocompatibility and proper antibacterial possessions, which could be a capable selection for prospective clinical usages in bone regeneration or as an approaching bone replacement biomaterial.^48^

###  Scaffold-based alginate- HAp in bone tissue engineering

 Alginate (Alg), an anionic polymer due to massive biomedical usage, is attaining significance chiefly in bone tissue regeneration due to its biocompatibility and gel-developing belongings.^41,49-54^ Alg, as a renowned drug carrier, is used to make wound dressing. Antimicrobial factors or drugs are laden into the hydrogel.^55^ Alg scaffolds are broadly utilized in bone and cartilage tissue engineering because of their chemical resemblance to the ECM. For example, Injectable Alg-based composite gel extra mixed with HAp and Gel microspheres (GMs) were cross-linked through in situ freedom of calcium cations. In addition, tetracycline hydrochloride (TH) was laden into GMs to enrich the bioactivity of platforms. The consequences indicated that HAp and GMs effectively enriched the mechanical strengths of gel platforms at strain from 0.1 to 0.5, which became stable in the gel system and reduced degradation, along with swelling percentage and gelation period. Results revealed that the HAp and GMs extra assimilated Alg-based gel platforms, particularly the one with 6% (w/v) HAp and 5% (w/v) GMs, had appropriate physical act and bioactive possessions, therefore offer a prospective chance to be utilized for bone regeneration. In mixture with TH, the gel showed an advantageous influence on osteoblast action, which demonstrated a capable future for the local cure of pathologies including bone damage.^56^ Recently, using MD stimulation, our research group found the addition of 10% HAp and tricalcium silicate to the sodium alginate hydrogel matrix produces a more compact, stable, less hydrated structure and positively affects scaffold mechanical for cell attachment.^15^ In a study, the hydrogel based on Alg and nHAp laden with phenolic refined extracts was synthesized from the aerial slice of Linum usitatissimum (LOH) as the bone regeneration platform. nHAp was made according to the wet chemical method/precipitation response and assimilated into Alg hydrogel as the packing through physical cross-linking. The prepared nanocomposite had a porous structure with organized pores. The average pore dimension was in the array of 100–200 µm and the sponginess variety of 80–90%. The LOH release assessment indicated that around 90% of the laden drug was released in 12 hours tailed by a continued release ended 48 h. The hemolysis initiation quantity exhibited that the prepared scaffolds were hemocompatible with insignificant hemolysis stimulation. The cell viability assay revealed the biocompatibility of the scaffolds, which encouraged proliferative influence in an amount reliant on routine. This work demonstrated the produced nanocomposites were osteoactive for bone regeneration usages.^57^ Aslam Khan et al fabricated polymeric nanocomposite through free-radical polymerization from sodium Alg, HAp, and silica with diverse GO contents ((sodium Alg)-g-(nHAp@SiO_2_@GO)). The porous platforms were prepared to utilize the freeze-drying method. They indicated diverse mechanical strengths as SAG-1 had the minimum compression strength and compression modulus 2.14 ± 2.35 and 16.51 ± 1.27 MPa. However, SAG-4 indicated supreme compression strength and compression modulus 13.67 ± 2.63 and 96.16 ± 1.97 MPa, respectively. Likewise, SAG-1 showed the minimum and SAG-4 indicated extreme apatite deposition, cell attachment, and cell proliferation. Therefore, the prepared nanocomposite could be a possible platform to restore cracked bone organs in bone regeneration.^58^

## Scaffolds-based synthetic polymer-HAp in bone tissue engineering

###  Scaffolds-based polycaprolactone - HAp in bone tissue engineering 

**Figure 2:**
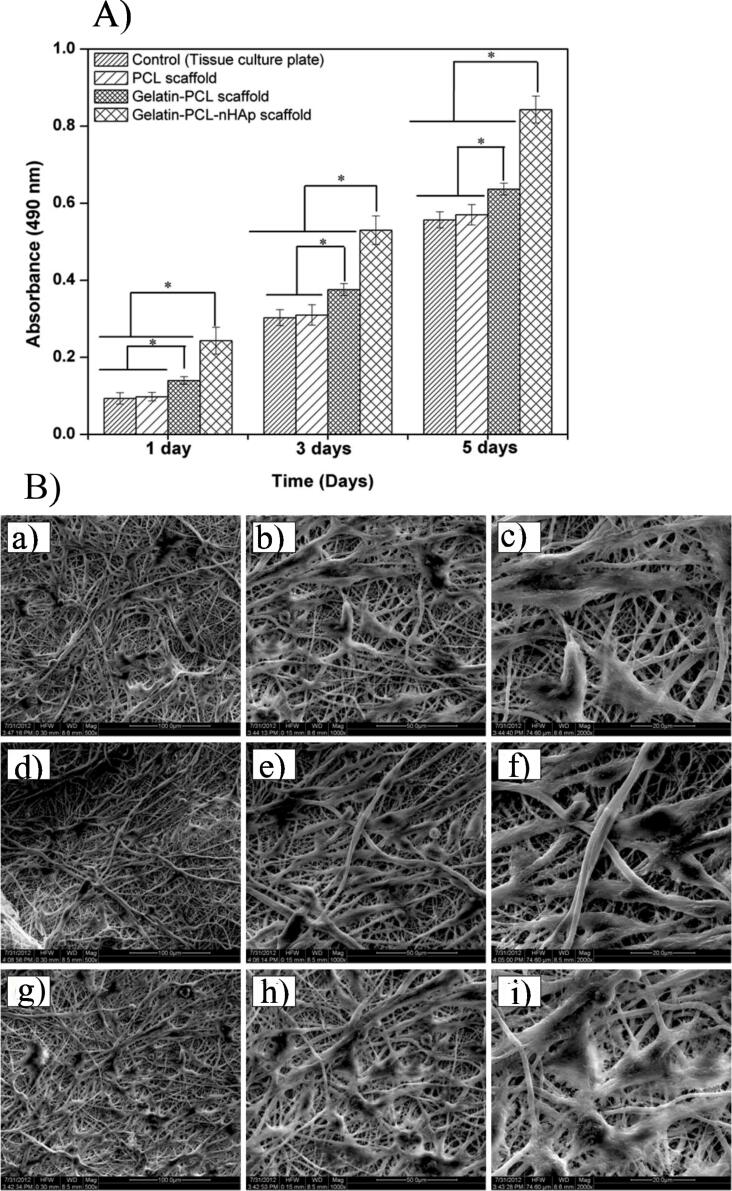
A) MTT assay of human osteoblasts on control (Tissue culture plate), PCL, Gel-PCL nanofibrous platform and Gel-PCL-nHAp - 20 min and B) Cell attachment and morphology of human osteoblasts on Gel-PCL-nHAp nanocomposite -20 min. Images a), b), and c) indicated the osteoblasts morphology later 1 day; images d), e), and f) later 3 days; images (g), (h), and (i) later 5 days of cell cultivation at 500 × , 1000 × , and 2000 × enlargement respectively. Later 5 days of cultivation time extreme surface on a platform enclosed by osteoblast cells. (* showed *P* < 0.05). Reproduced with permission from Gautam et al.^69^ Copyright Elsevier (2021)

 PCL is one of the greatest important synthetic polymers being studied for bone platform usage because of its mechanical strengths and outstanding biocompatibility to enhance cell proliferation and osteogenesis, which can be finally used for bone regeneration.^21,59-63^ In a study, HAp was assimilated on PCL-based 3D printed platforms at two diverse amounts, 80:20 and 60:40. Platforms were fabricated with two various combination techniques, solvent casting, and melt blending. Based on the results, the melt-blending-resulting platforms indicated more encouraging mechanical strengths, along with the assimilation of HAp. Moreover, the melt-blending-resulting scaffolds enhanced osteogenesis and in vitro cytocompatibility. Generally, PCL/HAp scaffolds were encouraging selections for bone regeneration, principally when fabricated by the MB technique.^64^ The assimilation of osteoconductive HAp into PCL might enrich the material hydrophilic behavior, protein adsorption, and subsequently, bone development. In a study, PCL/HAp scaffolds with 5, 10, and 25 wt% of HAp were fabricated by melt compounding tailed by hot compression. The incorporation of 5 and 10 wt% of HAp provided possessions analogous to the pure PCL; consequently, these compositions were selected to fabricate platforms by 3D printing technique. The prepared composites indicated outstanding printability and uniform distribution of the HAp. The compressive strength modulus of both compacted examples and platforms was about 30 MPa, resembling cancellous bone. The existence of enhancement of HAp amount joint with surface cure utilizing NaOH improved osteoblast growth.^65^ Cestari et al fabricated composites comprised ∽85 wt% PCL and ∽15 wt% nHAp from biogenic foundations and were 3D printed via an extrusion-founded procedure to attain spongy platforms appropriate for bone tissue engineering. The elastic modulus (177–316 MPa) originated to be in the array described for usual trabecular bones which was enhanced by the existence of the nHAp fragments. Furthermore, cell attachment, viability, and growth were mainly enhanced in the platforms comprising nHAp relative to PCL, the greatest consequences being discovered when mussel shell-resulting HAp was utilized. Generally, the consequences demonstrated that PCL/bio-HAp platforms indicated enriched mechanical strengths and improved bioactivity relative to neat PCL ones.^66^ A multifunctional scaffold founded on polylactic acid (PLA)/ PCL/ HAp nanocomposite comprising zinc oxide (ZnO) and Graphene (Gr) NPs was produced by solvent casting joint with die-cast methods as an implant in bone regeneration. It was estimated that the scaffold enclosed 1% ZnO and 1% Gr with Young’s modulus of 1540.5 ± 169.426MPa and the neat scaffold indicated Young’s modulus of 1194.81 ± 215.342MPa. The improvement in elongation at break was because of the existence of PCL in the polymer platform. The ideal group with 1% ZnO and 1% Gr showed antibacterial behaviors higher than other scaffolds. Likewise, the existence percentage of fibroblast cells in the neighborhood of the ideal matrix was meaningfully different from other groups. Based on the SEM results, the osteoblast cell was entirely flattened on the surface. The cell had a philopedia and was wholly attached to the exterior. Based on the results, the assimilation of the NPs enhanced the mechanical properties with enriched biological possessions and its anti-bacterial behavior provided this scaffold a capable selection for bone regeneration.^67^ Biodegradable PCL/HAp platforms were synthesized by the combination of PCL and HAp NPs through the melting deposition-forming technique. Based on the findings, PCL/HAp scaffolds showed suitable biodegradability and respectable biocompatibility, which could increase the MC3T3-E1 osteoblast cell growth. In addition, in vivo, tests were performed for the rats with skull flaws and rabbits with bone deficiencies. Consequently, the PCL/HAp composites endorsed the attachment and diffusion of bone cells, which assisted in the proliferation of bone cells and bone renovation. By a composite strategy to load doxorubicin (DOX) and attain continued drug release, 3D printed PCL/HAp/DOX composites could increase bone restoration and be anticipated to hinder possibly the tumor cells later bone tumor resection.^68^ In one study, an electrospun nanocomposite scaffold was synthesized by utilizing Gel, PCL, and nHAp for bone tissue engineering. The gel-PCL mixture was electrospun and then blended with nHAp (1 wt%) for various periods. The Gel-PCL-nHAp platform -20 min indicated the normal fiber diameter of 615 ± 269 nm and normal pore dimension of 4.7 ± 1.04 μm and similarly showed the existence of nHAp fragments ended the Gel-PCL scaffold external. MTT test indicated worthy viability and meaningful growth of human osteoblasts on Gel-PCL-nHAp nanocomposite (Figure 2A). Furthermore, cell-platform construction demonstrated well-organized cellular adhesion and sufficiently extended cells, and it similarly portrayed the typical polygonal structure of cells on the Gel-PCL-nHAp platform (Figure 2B). Consequently, the consequences of the in-vitro study of the electrospun platform confirmed that the Gel-PCL-nHAp platform could be a prospective selection for bone tissue engineering usages.^69^

###  Scaffolds-based poly (L-lactic acid) - HAp in bone tissue engineering 

**Figure 3:**
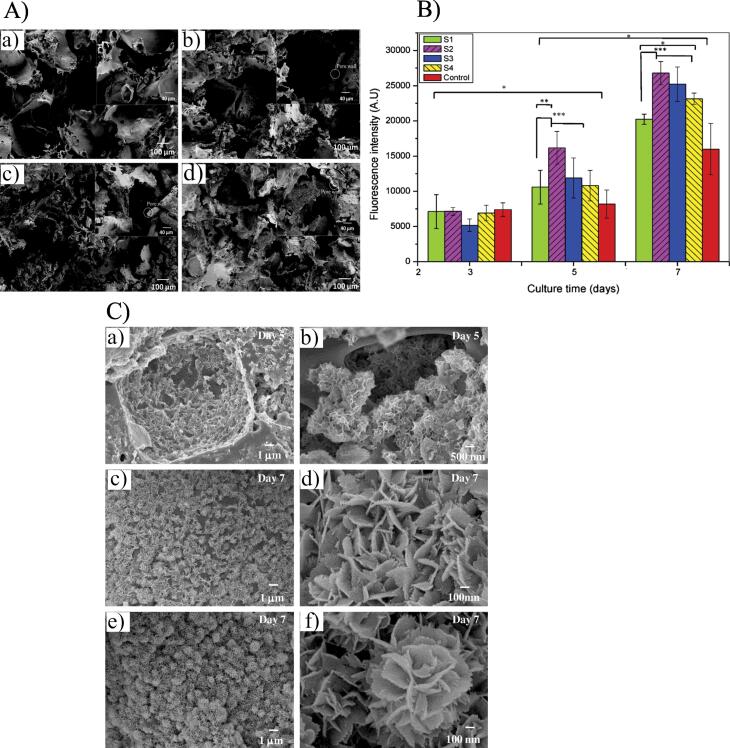
A) FE-SEM images of platforms, produced at various proportions of HAp to PDLLA: a) S1 PDLLA, b) S2 HAp/PDLLA, c) S3 HAp/PDLLA, and d) S4 HAp/PDLLA at the low enlargement of (200 × ), (S1: HAp mass: PDLLA mass 0: 0.455; S2: HAp mass: PDLLA mass 0.0455: 0.455; S3: HAp mass: PDLLA mass 0.091: 0.455; S4: HAp mass: PDLLA mass 0.136: 0.455), B) Cell viability and growth on the platforms later 3, 5, and 7 d of culture over alamarBlue test. All results were indicated as means ± SD; n = 6. *P < 0.001,**P < 0.01 and ***P < 0.05 (data compared with S1 PDLLA), and C) FE-SEM images of prepared HAp/PDLLA composite later absorption in SBF: a and b) at 5 days with enlargements of (5000 × ) and (10 000 × ), and c and d) at 7 days with enlargements of (5000 × ) and (50 000 × ) for S3 HAp/PDLLA composite; e and f) at 7 days with enlargements of (5000 × ) and (50 000 × ) for S4 HAp/PDLLA composite. Reproduced with permission from Nga et al.^73^ Copyright Elsevier (2015)

 PLA is a bio-degradable polymer and is generally utilized in bone tissue engineering applications. ^70-72^ Nga et al produced a synthesis of bio-imitative HAp nanorod/poly(d,l) lactic acid (HAp/PDLLA) platforms with the usage of solvent casting joint with a salt-leaching method for bone regeneration. Consequences indicated that HAp nanorod/ PDLLA platforms that imitate the construction of native bone were effectively fabricated. The prepared platforms showed macropore systems with great sponginess (80%–84%) and a mean pore dimensions array of 117–183 μm (Figure 3A). These platforms indicated outstanding apatite-developing aptitudes. The fast development of bone-similar apatites with flower-like morphology was perceived after 7 days of cultivation in simulated body fluids (SBFs). The platforms that indicated a greater ratio (30 wt.%) of HAp revealed superior cell attachment, growth, and spreading than those with a minor ratio of HAp as the days of cultivation enhanced (Figure 3B and 3C). This work indicated a resourceful method for emerging biomimetic scaffolds, which had latent uses in bone regeneration.^73^

 In one study, the composite of poly(trimethylene carbonate), PLA, and HAp (PTMC/PLA/HA and PTMC/HAp) were fabricated by the alteration and combination of PTMC with PLA and HAp, respectively. The PTMC/PLA/HAp and PTMC/HAp platforms were further produced by additive engineering utilizing the natural 3D printing technique with the PTMC/PLA/HAp and PTMC/HAp materials, respectively. The consequences showed that PTMC/PLA/ 25%HAp and PTMC/ 25%HAp platforms demonstrated low toxicity, suitable biodegradability, excellent biocompatibility, and increased osteoblast cell (MC3T3-E1) proliferation. Furthermore, PTMC/PLA/ 25%HAp and PTMC/ 25%HAp platforms improved the attachment and growth of MC3T3-E1 cells and supported the bone cell growth and stimulation of bone organ development. Consequently, PTMC/PLA/25%HAp composites revealed improved mechanical strengths, inferior cell cytotoxicity, and greater cell growth than PTMC/25%HAp composites.^74^ Bayart et al fabricated PLA and PLA/ HAp platforms (10 wt.%) by a fused filament fabrication (FFF)-derived technique. It was confirmed that the platforms were biocompatible and that cells achieved to adhered and grown. The consequences demonstrated that the existence of HAp in the platforms prompted a swifter and extra comprehensive biodegradation, with a steady reduction in the molar mass (Mn) and compressive strengths ending time. The mechanical strengths of the PLA_90_/HAp_10_ platforms were appropriate for bone rejuvenation usages, due to their sufficient compressive strengths that persisted great adequate. All the platforms were biocompatible and delivered cell growth. The Mn of PLA_90_/HAp_10_ platforms importantly decreased throughout conditioning, which was a suitable indication that this kind of substantial construction could biodegrade later insertion in the body, while neat PLA did not. This confirmed the significance of the incorporation of HAp into PLA to the extent of steady weight loss instantaneously with bone development in vivo. The ARBURG Plastic Freeforming (APF) method provided platforms with substantial properties from particularly prepared pellets deprived of the necessity to arrange 3D printing filament, evading extra weight loss of the formulation comprising Hap.^75^ In an investigation, two micron-sized HAp were examined for electrospinning with PLA at numerous amounts, spray-dried HAp (HAp1) and sintered HAp (HAp2) fragments. Scanning electron microscope (SEM) consequences indicated that fiber diameter and exterior coarseness of 15 and 20 wt% PLA fibers were meaningfully changed by the integration of any HAp. Weight loss percentages of HAp2-packed platforms in vitro over 14 days were more minor than for HAp1-packed platforms because of the enriched distribution of HAp2 inside the PLA environment and decreased hollows in PLA/HAp2 boundary. Lastly, the accumulative filler exterior area increased thermal steadiness as it decreased the thermal weight loss of the polymer.^76^ In a study, PLA was strengthened with HAp microparticles and strontium (Sr) through the melt extrusion/hot pressing engineering route to yield platforms with bone rejuvenation capacities. Based on the results, with the accumulation of HAp, the sponginess of the PLA-HAp platforms was decreased. However, the incorporation of Sr enhanced the porosity of the platforms, and this could probably be attributed to the grain improvement ability of Sr. The mechanical assessment results demonstrated that the insertion of Sr created the extremely normal Vickers hardness result of 49.1 HV. The prepared platforms indicated bioactivity abilities, consequently, they could aid as appropriate bone rejuvenation materials.^77^

###  Scaffolds-based Poly (vinyl alcohol) and poly (propylene fumarate) - HAp in bone tissue engineering 

**Figure 4:**
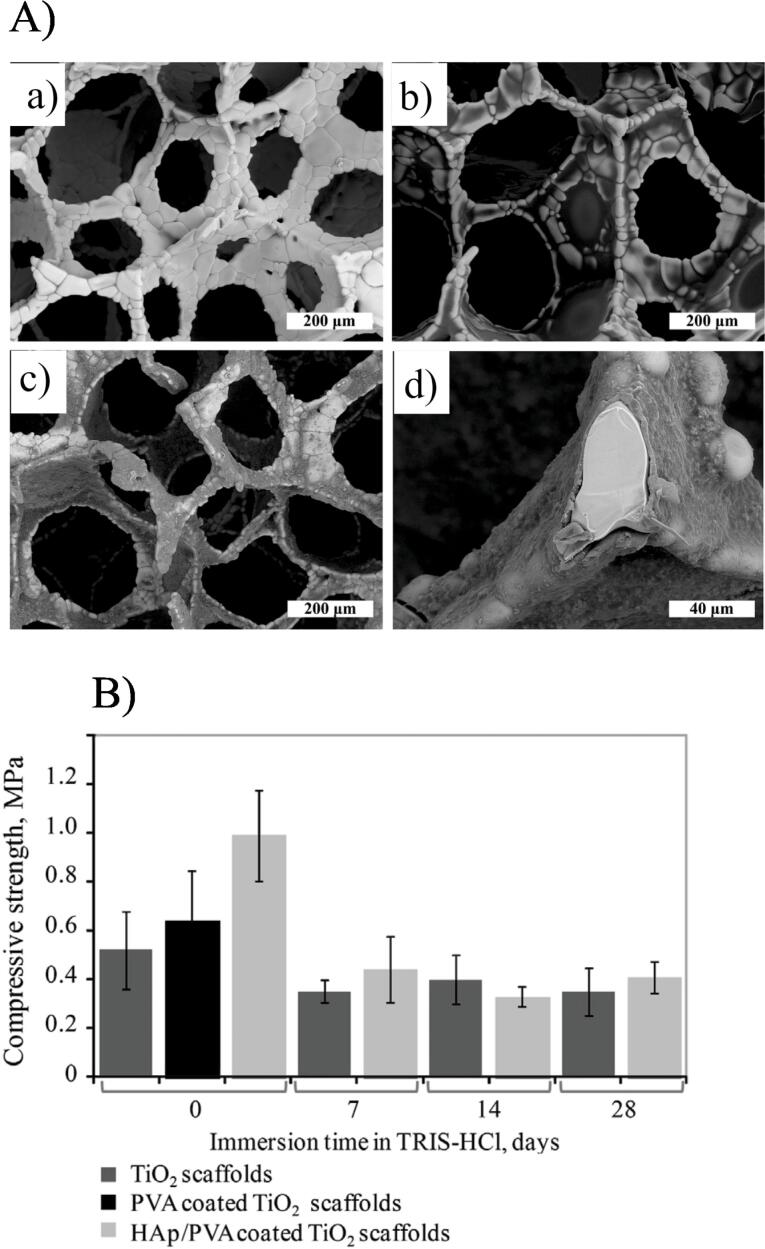
A) FE-SEM micrographs of a)TiO_2_ platform; b)PVA covered TiO_2_ platform, c) HAp/PVA covered TiO_2_ platform, and d) cross-sectional observation of HAp/PVA covered TiO_2_ and B) Compressive strength of TiO_2_ and HAp/PVA coated TiO_2 _platforms later in vitro eight loss assay. Reproduced with permission from Stipniece et al.^83^ Copyright Elsevier (2016)

 Poly(vinyl alcohol) (PVA) has been explored as a scaffold for tissue regeneration because of its outstanding biocompatibility, biodegradability, mechanical strength, and most prominently, due to its capability to be dissolved in aqueous solutions.^62,78-81^ In a study, a novel Alg- PVA- HAp hydrogel was prepared with optimum rheological belongings for 3D bioprinting of mouse calvaria 3T3-E1 (MC3T3) cells into platforms of extraordinary form fidelity. Degradation assays in α-MEM cell culture media indicated that the 3D-printed Alg-PVA-HAp platforms stayed intact for two weeks. 3D printed platforms with ideal formulations indicated adequate integrity and mechanical strengths ending a 14-day incubation time. MC3T3 cells were finely dispersed and compressed through the perfect hydrogel preparation and showed extraordinary viability over the achievement of the 3D printing progression. Therefore, the fabrication of this new and osteoconductive Alg-PVA-HAp preparation and its capability to 3D bioprint organ-engineered platforms provided it an encouraging selection for bone flaw curing.^82^ Stipniece et al fabricated macroporous TiO_2_ platforms with pore dimensions array from 100 to 500 μm were attained by the polyurethane foam repetition technique. Identical, a limited micrometers of tinny covering containing HAp combined in the existence of PVA were attained on the spongy TiO_2_ platforms over vacuum-aided impregnation technique whereas the unique macroporosity and open hole construction of the TiO_2_ platforms were kept (Figure 4A). The consequences demonstrated that a mixture of HAp/PVA nanocomposite coverings and spongy TiO_2_ ceramic provided a composite scaffold with enriched mechanical strengths by attaining the first mechanical strength up to 0.99 ± 0.19 MPa and heightened in vitro bioactivity (Figure 4B).^83^

 A new bioactive PVA/ Whey protein isolate (WPI)/ HAp composite platform with gentamicin (GEN)-laden at variable proportions was effectively produced by the 3D printing method. The 3D PVA/WPI/HA/12GEN platform was synthesized outstandingly with its 675 µm pore dimension. Compression studies indicated that the 3D composite possessed a compressive strength of 1.28–1.22 MPa and strain of % 12.89–8.70 and therefore encountered the mechanical properties of human trabecular bone. Furthermore, the compressive strength and strain results of the composites were reduced somewhat because of the addition of the GEN. It was perceived that the incorporation of GEN into the platform did not meaningfully influence the swelling and weight loss properties, and the composites degraded by around 55% on the 10th day. Assimilation of GEN to the solution containing PVA, WPI, and HAp induced an escalation in viscosity. The platforms demonstrated a regulated release pattern up to 240 and 264 h and were free with the Korsmeyer-Peppas kinetic model based on the maximum correlation quantity. Cell study indicated that biocompatible constructions were manufactured, and osteoblasts shaped filopodia extensions, contributing to vigorous cell adhesion. Based on the findings, a 3D GEN-laden PVA/WPI/HAp might be a favorable advance for bone flaw healing in bone regeneration usages.^84^ A novel sort of spongy PVA/sodium alginate/HAp (PVA/SA/HA) hydrogels with tunable construction and mechanical strengths were produced by the double-crosslinking technique. The PVA/SA/HAp hydrogels showed even, interpenetrating porous construction. The adjusted compression modulus (41.74 ± 7.86 kPa), moisture amount(86.99 ± 0.72%), and sponginess (79.98 ± 1.61%) of the scaffolds were attained through setting the weight relation of PVA/SA/HAp at 42:18:40. In vitro weight loss and mineralization of the prepared scaffolds confirmed that the hydrogels were slowly degraded in phosphate buffered solution (PBS) solution and sheet-similar HAp nanocrystals were simply shaped on the exterior. Additionally, cell culture consequences revealed that the PVA/SA/HAp scaffolds had no undesirable influences on MC3T3-E1 cell viability. The ALP activity expressions indicated that the nano-HAp particles combined with composite scaffolds noticeably enhanced the ALP activity of the cells. It revealed that the fabricated PVA/SA/HAp scaffolds could be a capable selection for bone renovation and bone regeneration.^85^ In a study, The scaffolds prepared by developing a gel (physically steadied) and successively dehydrated utilizing the lyophilization method obtainable morphologies with pore dimension in the individualities essential for cell growth. The platforms indicated a precise surface area that could provide the bioactivity of the polymers that produced the intended environments. The CS, CS-PCL, and CS-PCL-PVA-HAp platforms showed no cytotoxic result at 10 days assessed with the Alamar blueTM cellular test. The differentiation assay revealed the differentiation of swine dental pulp stem cells tailed by mineralization. The prepared platforms endorsed the attachment, viability, and growth of swine pulp stem cells and had a trivial consequence on mineralization capability in CS-PCL-PVA-HAp platforms.^86^

 Poly(propylene fumarate) (PPF) is a cross-linkable polymer intended for bone regeneration usage.^87,88^ PPF is an unsaturated copolyester founded on fumaric acid that was extensively utilized for tissue regeneration requests due to its tailorable mechanical strength and extraordinary biocompatibility.^89^ Li et al fabricated zinc-doped HAp powders with diverse Zn/(Zn + Ca) molar relations of 0, 0.025, 0.05, and 0.1 by a wet chemical technique. The resultant Zn-HAp was chosen as HAp, Zn2.5-HAp, Zn5-HAp, and Zn10-HAp. The Zn-HAp at 30 wt% were utilized to produce PPF-founded nanocomposite platforms (HAp/PPF, Zn2.5-HAp/PPF, Zn5-HAp/PPF, and Zn10-HAp/PPF). Live/dead assay indicated that these nanocomposites were biocompatible and endorsed outstanding attachment of MC3T3-E1 preosteoblast cells. ALP activity assessment and alizarin red staining indicated suitable osteogenesis and calcium deposits for MC3T3-E1 cells cultured on fabricated nanocomposites. Consequently, the prepared Zn5-HA/PPF nanocomposite was a talented platform for bone regeneration.^90^

## Conclusion

 In this review, the latest materials of practical HAp-based polymeric scaffolds in bone regeneration have been explored to provide the appropriate selection, intended as platforms with perfect possessions. The usage of HAp fragments as a significant construction provides a source for multifunctional HAp fragments which can fulfill the bone tissue microenvironment or stimulate increasing consideration. Taking into attention the prospect of mixing polymers and HAp, to adapt the belongings of the scaffolds, the features of the perfect biomaterial have been investigated to be afterward useful in the medicinal arena. HAp-based polymeric scaffolds, being an ECM-simulating biopolymer offer an appropriate condition for the loading of cells. Bone tissue engineering construction based on cell-laden HAp-based polymeric scaffolds will be a superior feature to study. The mixture of HAp with polymeric materials (e.g., CS, Gel, PLA, PCl, PVA, and PPF) can provide the enhancement of platforms’ bioactivity and applicability in bone tissue engineering. HAp-based polymeric scaffolds can increase cell-to-cell linking, differentiation, growth, and adhesion of the scaffolds. Moreover, the usage of nHAp likewise recovers the bone-developing ability and the mechanical strength of the freshly shaped bone.

## Competing Interests

 None to declare

## Consent for Publication

 Not applicable.

## Data Availability Statement

 No data is used in this review paper. All Permissions for figure reuse are granted from copyright holder.

## Ethical Approval

 Not applicable.
